# Lipoarabinomannan in sputum to detect bacterial load and treatment response in patients with pulmonary tuberculosis: Analytic validation and evaluation in two cohorts

**DOI:** 10.1371/journal.pmed.1002780

**Published:** 2019-04-12

**Authors:** Masanori Kawasaki, Carmenchu Echiverri, Lawrence Raymond, Elizabeth Cadena, Evelyn Reside, Maria Tarcela Gler, Tetsuya Oda, Ryuta Ito, Ryo Higashiyama, Kiyonori Katsuragi, Yongge Liu

**Affiliations:** 1 Otsuka Pharmaceutical Company, Tokyo, Japan; 2 Tropical Disease Foundation, Makati City, Metro Manila, Philippines; 3 Lung Center of the Philippines, Quezon City, Metro Manila, Philippines; 4 Jose R. Reyes Memorial Medical Center, Manila City, Metro Manila, Philippines; 5 The Medical City, Pasig City, Metro Manila, Philippines; 6 Otsuka Manila Research Center, Otsuka (Philippines) Pharmaceutical, Makati City, Metro Manila, Philippines; 7 Otsuka Pharmaceutical Development & Commercialization, Rockville, Maryland, United States of America; University of Cape Town, SOUTH AFRICA

## Abstract

**Background:**

Lipoarabinomannan (LAM) is a major antigen of *Mycobacterium tuberculosis* (MTB). In this report, we evaluated the ability of a novel immunoassay to measure concentrations of LAM in sputum as a biomarker of bacterial load prior to and during treatment in pulmonary tuberculosis (TB) patients.

**Methods and findings:**

Phage display technology was used to isolate monoclonal antibodies binding to epitopes unique in LAM from MTB and slow-growing nontuberculous mycobacteria (NTM). Using these antibodies, a sandwich enzyme-linked immunosorbent assay (LAM-ELISA) was developed to quantitate LAM concentration. The LAM-ELISA had a lower limit of quantification of 15 pg/mL LAM, corresponding to 121 colony-forming units (CFUs)/mL of MTB strain H37Rv. It detected slow-growing NTMs but without cross-reacting to common oral bacteria. Two clinical studies were performed between the years 2013 and 2016 in Manila, Philippines, in patients without known human immunodeficiency virus (HIV) coinfection. In a case-control cohort diagnostic study, sputum specimens were collected from 308 patients (aged 17-69 years; 62% male) diagnosed as having pulmonary TB diseases or non-TB diseases, but who could expectorate sputum, and were then evaluated by smear microscopy, BACTEC MGIT 960 Mycobacterial Detection System (MGIT) and Lowenstein-Jensen (LJ) culture, and LAM-ELISA. Some sputum specimens were also examined by Xpert MTB/RIF. The LAM-ELISA detected all smear- and MTB-culture–positive samples (*n* = 70) and 50% (*n* = 29) of smear-negative but culture-positive samples (*n* = 58) (versus 79.3%; 46 positive cases by the Xpert MTB/RIF), but none from non-TB patients (*n* = 56). Among both LAM and MGIT MTB-culture-positive samples, log_10_-transformed LAM concentration and MGIT time to detection (TTD) showed a good inverse relationship (r = −0.803, *p* < 0.0001). In a prospective longitudinal cohort study, 40 drug-susceptible pulmonary TB patients (aged 18-69 years; 60% male) were enrolled during the first 56 days of the standard 4-drug therapy. Declines in sputum LAM concentrations correlated with increases of MGIT TTD in individual patients. There was a 1.29 log_10_ decrease of sputum LAM concentration, corresponding to an increase of 221 hours for MGIT TTD during the first 14 days of treatment, a treatment duration often used in early bactericidal activity (EBA) trials. Major limitations of this study include a relatively small number of patients, treatment duration up to only 56 days, lack of quantitative sputum culture CFU count data, and no examination of the correlation of sputum LAM to clinical cure.

**Conclusions:**

These results indicate that the LAM-ELISA can determine LAM concentration in sputum, and sputum LAM measured by the assay may be used as a biomarker of bacterial load prior to and during TB treatment. Additional studies are needed to examine the predictive value of this novel biomarker on treatment outcomes.

## Abstract 2

Yongge Liu and colleagues develop a new immunoassay for measuring LAM in sputum and test whether it can be used for a biomarker for treatment response.

## Author summary

**Why was this study done?:**

- Currently, tuberculosis (TB) treatment is largely standardized, with fixed regimens and durations depending on known or estimated drug susceptibility of the isolates patients harbor. Treatment response monitoring can only be performed using sputum smear, which has low sensitivity and low specificity and is only semiquantitative, and sputum culture, which takes weeks to months for results and often is not performed in developing countries because of resource limitations.
- This approach is not ideal: on the one hand, patients with low bacterial burden are likely treated with unnecessarily long durations of toxic drugs, while on the other hand, patients with low-adherence and poor responders are not identified early on to prevent the development of drug resistance and transmission of TB to others.
- The lack of a real-time measure of bacterial burden also prevents innovative design of clinical trials to accelerate TB drug and regimen development.

**What did the researchers do and find?:**

- We isolated novel monoclonal antibodies against a major TB antigen, lipoarabinomannan (LAM), and developed an immunoassay (LAM-ELISA).
- The performance of the LAM-ELISA was evaluated in two clinical studies. We found that sputum LAM concentrations measured by the LAM-ELISA correlate with bacterial burden determined by culture prior to treatment. More importantly, sputum LAM concentration changes during TB treatment correlate with changes of bacterial burden measured by culture.

**What do these findings mean?:**

- Sputum LAM concentration is a measure of bacterial burden of pulmonary TB patients and may be a biomarker of response to treatment.
- Because LAM concentrations can be measured in hours by this assay versus weeks or even months by culture, personalized TB treatment and management may be possible by using this biomarker. Additionally, this biomarker may allow innovative clinical trial designs in TB drug or regimen development to shorten timeline and reduce cost.
- Newer immunoassays are being developed using the same antibodies in the LAM-ELISA that require minimum manual processing and can provide LAM concentrations in less than one hour. Such tests may be used in a point-of-care setting.

## Introduction

Tuberculosis (TB), caused by infection with *M*. *tuberculosis* (MTB), now ranks alongside human immunodeficiency virus (HIV) as a leading cause of death worldwide because of infection [1]. In contrast to the treatment management of viral diseases such as HIV and hepatitis C, in which real-time viral load tests have significantly improved disease management and treatment outcomes [2,3], current tools to measure MTB load are limited by either low sensitivity and low quantitative characteristics (sputum smear microscopy) or requiring up to two months to obtain results (sputum culture) [4]. Knowledge of bacterial load prior to and during treatment is important for the determination of infectiousness and, most importantly, whether a patient is responding to treatment. The lack of a real-time test to monitor treatment response impedes the improvement of current programmatic TB treatment, under which TB patients are treated largely in a standardized approach with fixed-duration-based regimens [5,6]. Inevitably, good responders are treated longer than necessary by drugs associated with significant adverse reactions, resulting in wasting resources (economic and human), while poor responders are not identified early, resulting in infection of others and potential development of drug resistance. Additionally, the lack of a real-time test to determine bacterial load limits the ability to conduct adaptive trial designs in TB drug development because culture results are the only accepted surrogate marker for efficacy [7].

Lipoarabinomannan (LAM) is a major component of the MTB cell wall, unique to *Mycobacterium* species, and has been considered as an ideal candidate for an antigen-based test [8]. Three studies published between the years 1990-2000 reported moderate sensitivity and high specificity using immunoassays based on various combinations of anti-LAM antibodies [9–11], but these studies only examined a limited number of samples, and neither follow-up large-scale studies nor further development of tests have been disclosed. One anti-LAM antibody-based immunoassay was commercialized (Clearview TB ELISA [enzyme-linked immunosorbent assay]), but this test was shown to have low specificity when using sputum because of cross-reaction with oral organisms [12,13].

The LAM molecule can generally be described as having three distinct structural domains: a phosphatidylinositol anchor, a mannan backbone, and an arabinan chain containing multiple arabinofuranoside (Ara*f*) residues with tetra- (Ara4) and hexa-Ara*f* (Ara6) termini that are capped by various carbohydrate motifs [14]. Since sputum inevitably contains oral bacteria—including actinomycetes, of which order the genus *Mycobacterium* is a subset—that produce LAM-like polymers [15] and *Mycobacterium* is a large family of various species including MTB, an MTB-specific test that uses sputum as the specimen would need antibodies targeting MTB-specific epitopes on the LAM. A large body of evidence indeed suggests species-unique characteristics of LAM. Rapid-growing nontuberculous mycobacteria (NTM) have uncapped ends or inositol phosphate caps (PILAMs) [16,17], while MTB and slow-growing NTM such as *M*. *avium* and *M*. *kansasii* are capped with one to three α-1,2-linked mannopyranose (Man) residues, resulting in ManLAM [16,17], with a dimannosyl unit as the major capping motif (80%) [18]. The mannoside caps of ManLAM in MTB may be further substituted with a unique methylthio-D-xylose (MTX) residue [17]. The MTX substitution has been identified in all MTB isolates analyzed to date, and a five-gene cluster dedicated to the biosynthesis of the MTX capping motif of MTB LAM has been identified [19]. Chan and colleagues indeed demonstrated that it is possible to develop antibodies against MTB-specific ManLAM regions to improve specificity [20].

To improve the performance of LAM-based sputum tests, we isolated three monoclonal antibodies using phage display technology. A recent study has demonstrated that two of the antibodies target the Man- and MTX-capped LAM [21], indicating an immunoassay using these antibodies may be specific for LAM from MTB. Here, we present the results from evaluation of the performance of an ELISA constructed with the three antibodies in preclinical studies and sputum specimens from pulmonary TB patients prior to and during treatment and examine the potential of using sputum LAM as a biomarker of bacterial load.

## Methods and materials

### Ethics statement

The animal experiments were performed in compliance with the Guidelines for Animal Care and Use of the Animal Care and Use Committee of Otsuka Pharmaceutical Company. Clinical study protocols with analysis plans and informed consent forms (ICFs) were approved prior to the start of the clinical studies by the following local Institutional Review Boards in Manila, Philippines: Tropic Disease Foundation Institutional Review Board, Lung Center Ethics Review Committee, Jose R. Reyes Memorial Medical Center Institutional Review Board, and The Medical City Institutional Review Board. Each study participant reviewed and signed the ICF.

### Purified LAM and bacterial strains or cell lines

Purified LAM from MTB Aoyama B was obtained from Nacalai Tesque, Kyoto, Japan. All bacterial strains and cell lines were obtained from the American Type Culture Collection (ATCC, Manassas, Virginia, USA).

### Preparation and characterization of anti-LAM monoclonal antibodies

Three monoclonal antibodies (S4-20, G3, and TB) were isolated using phage display technology. See a brief summary of the experimental procedures on the identification of anti-LAM monoclonal antibodies in S1 Appendix.

Antibodies “S4-20” and “TB” were obtained from rabbits, and “G3” was from chicken. The configuration using antibodies “S4-20” and “G3” as capture and antibody “TB” as detection demonstrated the highest signal from 38 clinical MTB isolates in the screening panel than any other combinations of the three antibodies and was therefore utilized as the final configuration for a 96-well format ELISA (LAM-ELISA hereafter). See S1 Appendix for the preparation of LAM-ELISA plates.

The LAM epitopes of the three antibodies were elucidated after the completion of the studies reported here. The work that led to the epitope identification is reported in a separate study [21], and the epitope information is summarized in Table 1.

**Table 1:** Antibodies used in the LAM-ELISA and their epitope specificities.

Role in the LAM-ELISA	Antibody Name	Epitope Specificity	Comments
Capture antibody	S4-20	MTX-capped Man2 motif	Specific for MTB and *M*. *avium* and *M*. *kansasii*
	G3	Unmodified Man2 motif	Specific for MTB and slow-growing NTM
		MTX-capped Man2 motif	
		Penta-mannose core	Importance for binding unknown
Detection antibody	TB	Man1- and Man2-Ara4	Specific for MTB and possibly slow-growing NTM
		Man2-Ara6	
		MTX-capped Man2-Ara	
		Uncapped Ara structures and PI-Ara4	

For details, see reference [21]. **Abbreviations**: Ara, arabinoside; ELISA, enzyme-linked immunosorbent assay; LAM, lipoarabinomannan; Man, mannopyranose; Man1: one mannopyranose residue; Man2: two α-1,2-linked mannopyranose residues; MTB, *M*. *tuberculosis*; MTX, methylthio-D-xylose; NTM, nontuberculous mycobacteria; PI-Ara4, phosphatidylinositol-capped Ara4; TB, tuberculosis.

### Extraction of LAM for measurement

To maximally expose the LAM antigen from bacilli for detection, various methods of extraction were evaluated, resulting in adoption of the following method for LAM extraction: adding 0.2 mL of 1.2 M NaOH solution to 0.4 mL of the sample (bacterial cell culture or sputum), heating the mixture at 100°C for 20 min, followed by adding 0.09 mL of 5 M NaH_2_PO_4_. These LAM extracts were then stored at −70°C until measured by LAM-ELISA.

To determine whether LAM epitopes might be affected by this extraction method, purified LAM was subjected to extraction, and LAM-ELISA readings were compared with LAM solutions without such an extraction treatment. As shown in Table A in S1 Appendix, this extraction method did not affect the measurements of purified LAM by the LAM-ELISA.

### LAM measurement by the LAM-ELISA

LAM measurement was conducted by adding LAM extracts (0.1 mL) to the LAM-ELISA plate. Following three washing steps, 40 ng/mL of biotin-conjugated detection antibody (0.1 mL) was added, followed by horseradish-peroxidase-conjugated streptavidin. 3,3′,5,5′-tetramethylbenzidine dihydrochloride and hydrogen peroxide were then added to each well. Color development was stopped by adding sulfuric acid and the optical density (OD) at 450 nm (using OD at 650 nm as the background) was measured by a microplate reader. A set of standards with known LAM concentrations was included in each plate to generate a standard curve for quantification of LAM.

### Analytic validation of the LAM-ELISA

Lower limit of quantitation (LLoQ) and limit of detection (LoD) were determined by measurement precision using serial dilutions of LAM extracts obtained from *M*. *bovis* Bacillus Calmette-Guérin Tokyo (BCG) culture. Linearity of LAM concentration and colony-forming units (CFUs) were determined using MTB strain H37Rv (H37Rv) by plating culture dilutions on Middlebrook 7H11 agar (Becton Dickinson, Franklin Lakes, NJ, USA) for CFU counts and by the LAM-ELISA for LAM concentration. The reactivity of the LAM-ELISA against 18 strains of NTM was evaluated using the same procedure. Specificity was examined against gram-positive and -negative bacterial cultures, including common oral bacteria. Interference was examined by comparing LAM concentrations with or without adding drugs for TB, pneumonia, and HIV to the sample prior to LAM extraction.

### Evaluation of clinical performance of the LAM-ELISA

Two clinical studies were performed in Manila, Philippines from 2013 to 2016. Studies were performed in accordance with the Good Clinical Practice guidelines of the International Conference on Harmonization. Handling of sputum specimens followed the local standard biosafety regulations.

**Fig 1:**
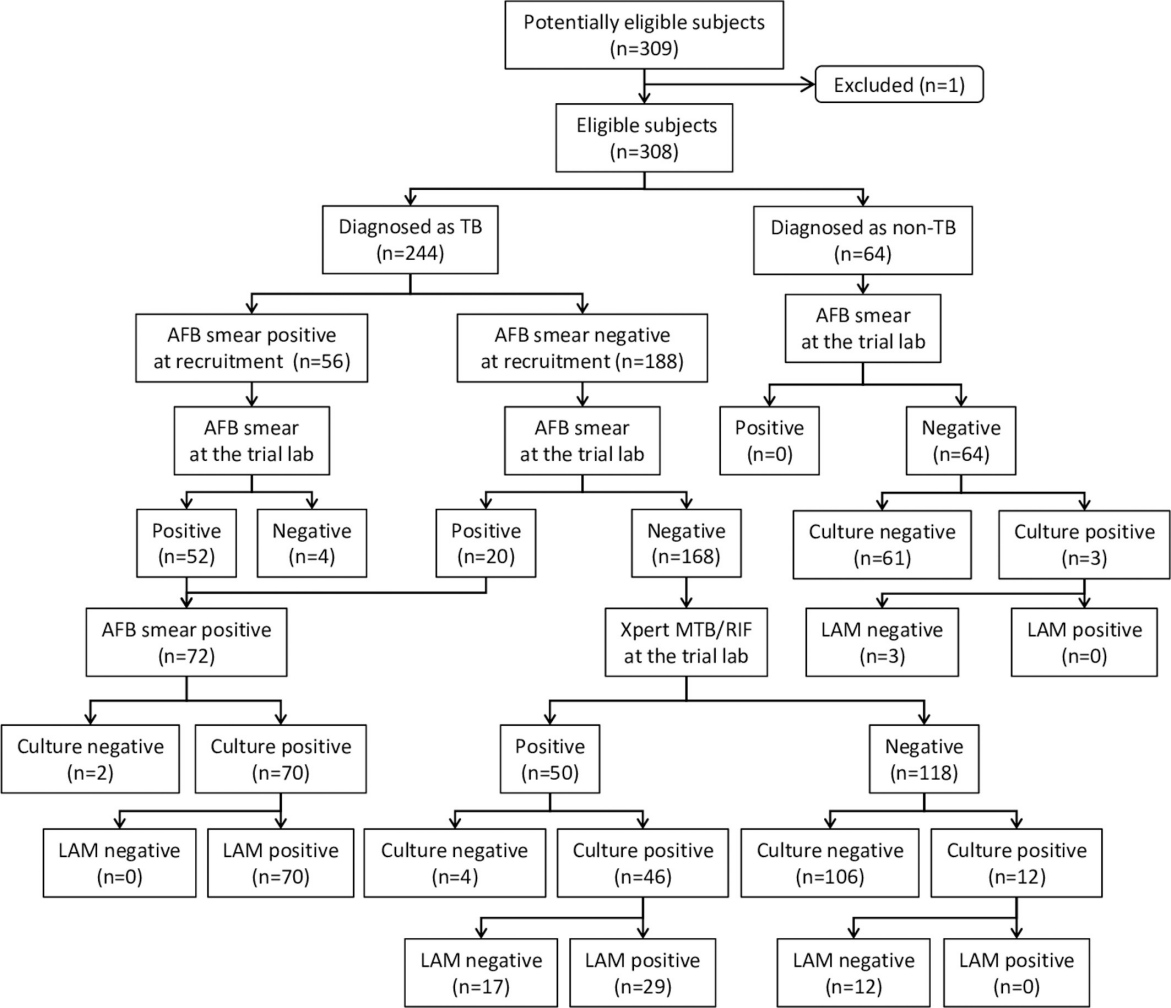
Study 1 patient enrollment scheme and sputum specimen microbiological characteristics. Patients were consecutively enrolled into two groups: diagnosed as TB or non-TB. Each patient provided one sputum specimen after enrollment. Note that 20 patients who were originally smear negative at recruitment were later determined as smear positive at the trial laboratory and reassigned as smear positive in final categorization. AFB, acid-fast bacillus; LAM, lipoarabinomannan; MTB, *M*. *tuberculosis*; TB, tuberculosis.

The first clinical study (Study 1) was to compare the sensitivity and specificity when using sputum LAM to diagnose TB with other standard detection methods (smear microscopy and culture) on a spectrum of specimens from patients with TB from disease-free to severe disease. To achieve this goal, a prospective case-control cohort diagnostic study was designed by enrolling patients without known HIV infection. The patient enrollment scheme and sputum specimen microbiological characteristics are shown in Fig 1.

Patients diagnosed as TB were categorized into two subgroups: acid-fast bacillus (AFB) direct smear microscopy (AFB smear) positive pulmonary TB and AFB smear negative but clinically diagnosed as pulmonary TB by physicians based on signs and symptoms including chest radiograph. Non-TB patients were those diagnosed with respiratory disease other than TB but who could expectorate sputum specimens. Both TB and non-TB patients were enrolled from a TB clinic, two general hospitals, and a lung specialty hospital.

Data were analyzed based on three categories of sputum specimens, respectively: 1) AFB smear and culture positive, 2) AFB smear negative but culture positive, and 3) non-TB (no mycobacteriology and chest radiograph evidence of active TB disease: chest radiograph, AFB smear, Lowenstein-Jensen [LJ] culture, and MGIT [BACTEC MGIT 960 Mycobacterial Detection System] culture all negative). Each patient provided one specimen. Patients were consecutively enrolled until at least 50 specimens were reached in each category to provide sufficient statistical power [22].

All sputum specimens were transferred to the trial laboratory (Tropic Disease Foundation [TDF], Manila, Philippines) and examined by LAM-ELISA, AFB smear, and LJ and MGIT culture, as described in Fig A in S1 Appendix. The staff performing the LAM-ELISA were unaware of the results from other tests or any information about the patients providing the sputum. Culture positivity was determined as MTB positive on either LJ or MGIT culture. Sputum specimens from patients with negative smear but who were diagnosed as TB were also examined with Xpert MTB/RIF (Cepheid, Sunnyvale, CA, USA), which has been shown to have more than 70% sensitivity than the MGIT culture [23,24]. The use of the Xpert MTB/RIF was to stop the enrollment after Xpert MTB/RIF positive samples reached 50 but before culture results were known.

Microbiological examinations are briefly described as follows: first, collected sputum specimen was examined by AFB smear microscopy. Then, the tube containing the sputum was stirred vigorously, followed by sitting still for 15 min. An aliquot of 0.4 mL of the sputum was used to prepare a LAM extract. To the rest of the sputum, an equal volume of freshly prepared N-acetyl-L-cysteine-sodium hydroxide solution (NALC-NaOH) was added. After 15 min of incubation with mixing, phosphate buffer (pH 6.8) was added to make the volume to 50 mL. The mixture was centrifuged for 15 min at 3,000 × *g*. The resultant supernatant was discarded, and the pellet was resuspended in a final volume of 2.5 mL phosphate buffer (pH 6.8), of which 0.2 mL was used for LJ culture, 0.5 mL for MGIT culture, and 0.5 mL for the Xpert MTB/RIF. Identification of MTB was performed by AFB staining and MGIT TBc Identification Test (TBcID; Becton Dickinson), using the positive MGIT culture or the positive LJ culture if the MGIT culture was contaminated. Contamination was determined based on growth on a blood agar plate. In cases in which the TBcID result was negative but AFB staining was positive and no contamination was detected, GenoType Mycobacterium CM and AS (HAIN Lifescience, Nehren, Germany) were used for NTM speciation.

**Fig 2:**
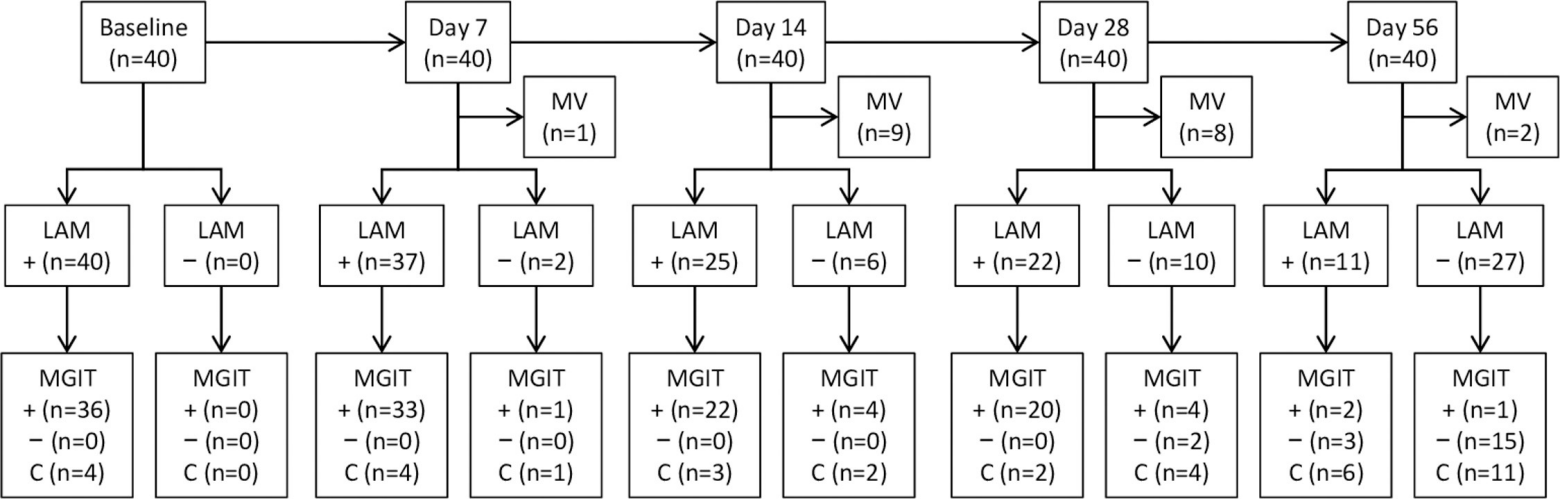
Study 2 patient enrollment scheme and LAM and MGIT results on sputum specimens collected during the 56-day treatment. MV: patients who were considered as missing since the visit dates were outside of the defined range of each visit date (see Materials and methods section for more details); Under LAM, +: LAM positive; −: LAM negative. Under MGIT, +: MGIT positive with MTB complex; −: MGIT negative; C: contaminated on MGIT. LAM, lipoarabinomannan; MGIT, BACTEC MGIT 960 Mycobacterial Detection System; MTB, *M*. *tuberculosis*; MV, missed visit.

To investigate whether sputum LAM can be used as a biomarker of bacterial load change during treatment, we conducted the second clinical study (Study 2) to evaluate the changes of sputum LAM concentrations during treatment up to 56 days and the relationship to the changes from MGIT time to detection (TTD). This prospective longitudinal cohort study consecutively enrolled 40 pulmonary smear-positive TB patients without known HIV coinfection or previous TB treatment. See the patient enrollment scheme and sample characteristics in Fig 2.

These patients received the standard 4-drug (isoniazid, rifampin, pyrazinamide, and ethambutol) treatment under directly observed therapy (DOT). Each of the 40 patients provided one sputum specimen prior to the start of treatment and at days 7, 14, 28, and 56 at the local health centers. Sputum specimens were then transferred to the trial laboratory (TDF) and examined by LAM-ELISA, AFB smear, MGIT culture, and Loopamp MTBC Detection Kit (TB-LAMP, Eiken, Tokyo, Japan) (a manual nucleic acid amplification test) as described in Fig B in S1 Appendix. The staff performing the LAM-ELISA were unaware of the results from other tests or any information about the patients providing the sputum. Each collected sputum specimen was decontaminated in a similar manner as in Study 1. Prior to centrifugation, 2.0 mL of the decontaminated specimen was taken out, of which 0.4 mL and 0.06 mL were used for LAM extraction and TB-LAMP detection, respectively. The rest of the specimen was then centrifuged, and the resultant pellet was resuspended in a final volume of 1.5 mL phosphate buffer (pH 6.8), of which 0.5 mL was used for MGIT culture. Because of some patients being unable to visit the site on the exact required dates during treatment, the days on treatment were calculated from the start of treatment with various ranges: day 7, between day 5 and 9 (day 7 ± 2); day 14, between day 11 and 17 (day 14 ± 3); day 28, between day 23 and 33 (day 28 ± 5); and day 56, between day 49 and 63 (day 56 ± 7). Data collected outside of these ranges were not included in the analysis.

Since the first human efficacy study during the development of a new drug is often to examine early bactericidal activity (EBA), a regulatory-agency-accepted efficacy endpoint [25,26] calculated as a decrease of sputum bacterial load from baseline after a short period of treatment (up to 14 days when a monotherapy is used), we compared the EBAs estimated by sputum LAM and culture in Study 2 at day 14 of treatment from the baseline. The EBA comparison was based on MGIT TTD changes because recent studies have suggested that an increased TTD from MGIT culture may substitute for the decline of CFU counts on solid media that is often used in traditional EBA studies [27,28].

AFB smear, LJ and MGIT culture, the Xpert MTB/RIF, the TB-LAMP, the MGIT TBc Identification Test, and the GenoType Mycobacterium CM and AS were performed according to internationally accepted procedures or manufacturer-provided instructions.

### Data analysis

Data were analyzed using SAS 9.4 (SAS Institute, Cary, NC, USA). Analytic validation and clinical studies were analyzed with prospectively planned analysis plans (see S2 Appendix) with additional post hoc analyses. In the nonclinical analytic validation studies, the relationship between log_10_-transformed LAM concentration and CFU counts was analyzed by calculating the Pearson correlation coefficient (r). For sensitivity and specificity parameters in Study 1, a 95% confidence interval (CI) was estimated by using the Wilson score confidence interval method and the Clopper Pearson interval method for a binominal proportion. Sensitivity was compared using the McNemar test. We also performed a post hoc Receiver Operating Characteristic (ROC) analysis to evaluate optimal cutoff for LAM. To this end, the Youden’s J index was calculated for the LAM-ELISA as “J = Sensitivity + Specificity − 1”. Relationships between LAM concentration and AFB smear score or MGIT TTD in Study 1 and Study 2 were analyzed post hoc by calculating the Pearson correlation coefficient (r) to examine the ability of sputum LAM concentration to bacterial load. In the correlation analysis, LAM and MGIT TTD were log_10_-transformed. A descriptive analysis of the EBA as measured by sputum LAM concentration and MGIT TTD was also performed post hoc.

## Results

### Analytic validation of the LAM-ELISA

**Fig 3:**
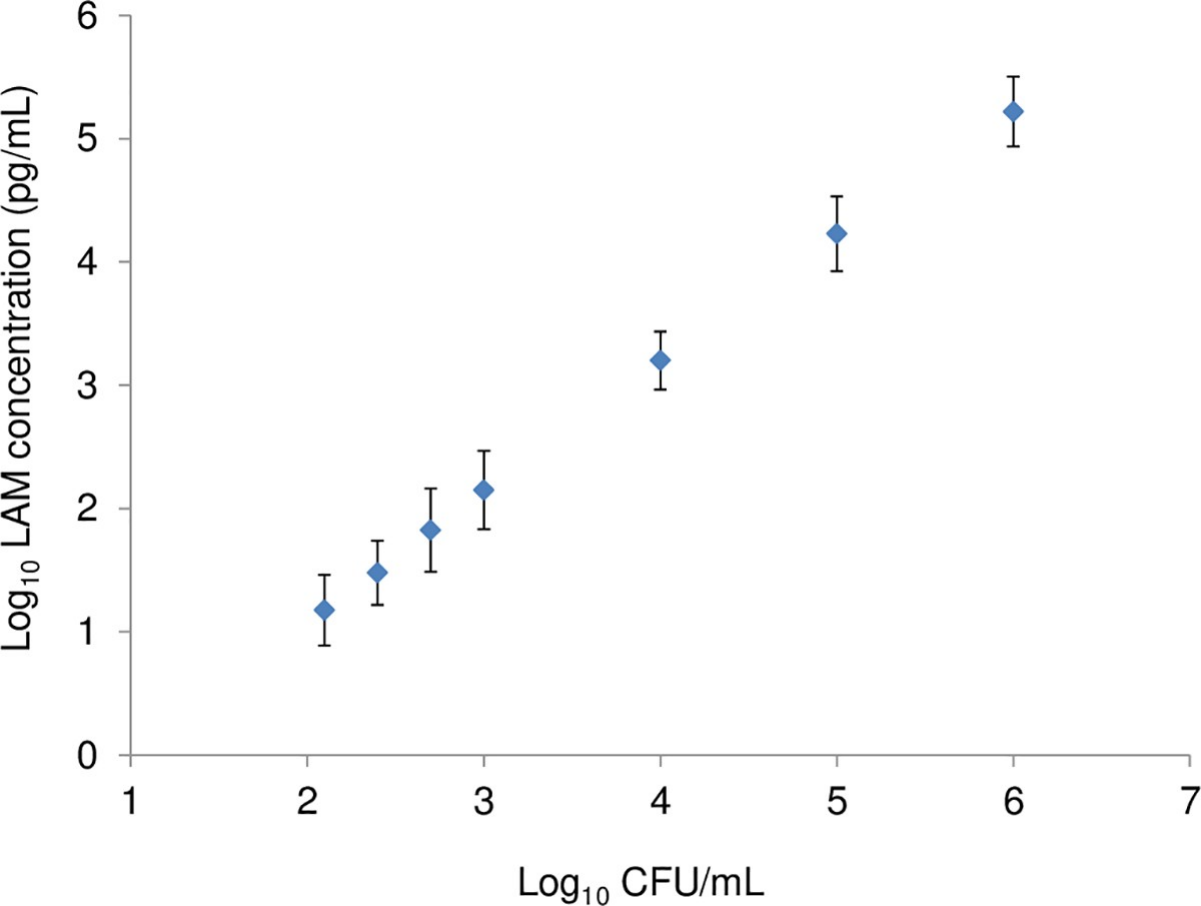
Correlation of LAM concentration and CFU counts in MTB H37Rv. Three independent cultures of MTB H37Rv were prepared in Middlebrook 7H9 broth medium. Aliquots of these cultures were frozen, and the CFU count of each frozen stock was determined. An aliquot of each frozen stock was thawed and diluted to achieve concentrations of 1.0 × 10^6^, 1.0 × 10^5^, 1.0 × 10^4^, 1.0 × 10^3^, 500, 250, and 125 CFU/mL. These dilutions were used to prepare for LAM extracts, and LAM concentration of each dilution was determined. This procedure was repeated for the other two independent cultures. The averages and their standard deviations of LAM concentration of each dilution among the three cultures were calculated and plotted in the figure. CFU, colony-forming unit; LAM, lipoarabinomannan; MTB, *M*. *tuberculosis*.

The LAM-ELISA demonstrated high reproducibility in intraday, interday, interoperator, and interlots from 12 repeated measures with a coefficient of variance (CV) of 5.1% or less. The LoD and LLoQ of the LAM-ELISA for TB detection were determined as 8.5 pg/mL and 15 pg/mL based on measurement precisions of serial dilutions of known LAM extracts from BCG culture for 18 times using 30% and 15% CV [29,30], respectively. Quantitatively, LAM concentration was linearly correlated to CFU counts of H37Rv (r = 0.986; CI: 0.963-0.994; *p* < 0.0001) as shown in Fig 3, with 1 pg/mL of LAM equaling about 8.06 CFU/mL of H37Rv.

The inclusivity of the LAM-ELISA against 18 NTM strains was determined using the LoD of 8.5 pg/mL as the cutoff. As shown in Table 2, the LAM-ELISA did not detect rapid growers at the highest concentration tested (1.0 × 10^6^ CFU/mL), except for *M*. *peregrinum*, for which a LAM concentration of 58.2 pg/mL was obtained at 1.0 × 10^6^ CFU/mL. On the other hand, the LAM-ELISA detected slow growers with variable sensitivities: the sensitivity for *M*. *kansasii*, *M*. *scrofulaceum*, and *M*. *haemophilum* may be higher than that for MTB but is generally lower for other slow growers.

**Table 2:** LAM concentration in NTM culture solutions determined by LAM-ELISA.

Category	Strain	CFU/mL				
		1.0 × 10^6^	1.0 × 10^5^	1.0 × 10^4^	1.0 × 10^3^	1.0 × 10^2^
Slow-growing mycobacteria	*M*. *avium* ATCC35712	27,406.0	2,246.9	245.6	19.0	<LoD
	*M*. *avium* ATCC700898	39,440.0	3,706.2	327.6	36.4	<LoD
	*M*. *intracellulare* ATCC15984	36,848.2	4,233.7	363.4	38.8	<LoD
	*M*. *kansasii* ATCC35775	4,990,264.0	572,184.8	96,505.6	1,154.5	65.9
	*M*. *simiae* ATCC25725	22,034.4	2,033.1	548.7	40.7	<LoD
	*M*. *scrofulaceum* ATCC19981	100,891.0	11,070.8	1,038.2	108.7	19.6
	*M*. *haemophilum* ATCC29548	Not tested	770,771.7	78,242.5	9,605.5	578.8
	*M*. *malmoense* ATCC29571	168,280.8	16,036.3	1,492.9	182.8	<LoD
	*M*. *shimoidei* ATCC27962	30,768.8	3,121.0	337.9	23.0	<LoD
	*M*. *szulgai* ATCC23069	<LoD	<LoD	<LoD	<LoD	<LoD
	*M*. *ulcerans* ATCC19423	25,258.6	2,437.6	289.7	<LoD	<LoD
	*M*. *marrinum* ATCC927	43,208.6	4,414.1	385.3	30.5	<LoD
Rapid-growing mycobacteria	*M*. *fortuitum* ATCC9820	<LoD	<LoD	<LoD	<LoD	<LoD
	*M*. *abscessus* ATCC19977	<LoD	<LoD	<LoD	<LoD	<LoD
	*M*. *aurum* ATCC23366	<LoD	<LoD	<LoD	<LoD	<LoD
	*M*. *smegmatis* ATCC19420	<LoD	<LoD	<LoD	<LoD	<LoD
	*M*. *chelonae* ATCC19235	<LoD	<LoD	<LoD	<LoD	<LoD
	*M*. *peregrinum* ATCC700686	58.2	<LoD	<LoD	<LoD	<LoD

All strains were cultured in Middlebrook 7H9 broth medium, and their aliquots were stored frozen. The CFU count of each frozen stock was determined. An aliquot of each frozen stock was thawed and diluted to achieve concentrations from 1.0 × 10^6^ to 1.0 × 10^2^ CFU/mL. These dilutions were used to prepare for LAM extracts, and LAM concentration of each dilution was determined. LoD: 8.5 pg/mL. **Abbreviations**: ATCC, American Type Culture Collection; CFU, colony-forming unit; ELISA, enzyme-linked immunosorbent assay; LAM, lipoarabinomannan; LoD, limit of detection; NTM, nontuberculous mycobacteria.

Forty-three microbial species from general bacteria in the oral cavity and pathogenic bacteria for pneumonia were examined for cross-reactivity. The LAM-ELISA cross-reactivity was not observed at up to 1.0 × 10^8^ CFU/mL with 39 common oral microbial species (Table B in S1 Appendix). No cross-reactivity was observed at up to 6.25 × 10^6^ CFU/mL for *Nocardia asteroides*. For three strains of the Chlamydia family, no cross-reactivity was observed at 1.0 × 10^3^ inclusion-forming unit/mL, the maximal achievable concentration in culture. We also showed that the performance of the LAM-ELISA was not affected by 29 potentially interfering substances: nine anti-TB drugs, nine antipneumonia drugs, nine anti-HIV drugs, mucin, and serum, at a final concentration of 200 μg/mL (except for mucin, which was at 2,000 μg/mL) (Table B in S1 Appendix).

### Performance of the LAM-ELISA on clinical samples

The baseline characteristics of the enrolled patients in the two clinical studies are summarized in Table 3.

**Table 3:** Baseline patient characteristics for Study 1 and Study 2.

Patient characteristics		Study 1				Study 2
		Total	Smear* positive	Smear* negative^	Non-TB	
						(*n* = 40)
		(*n* = 308)	(*n* = 76)	(*n* = 168)	(*n* = 64)	
Age (years)	Median	40	37	41	40	39
	Range	17–69	17–60	18–69	18–67	18–69
Sex	Male	191	51	100	40	24
		(62.0%)	(67.1%)	(59.5%)	(62.5%)	(60.0%)
	Female	117	25	68	24	16
		(38.0%)	(32.9%)	(40.5%)	(37.5%)	(40.0%)

*Smear results at the trial laboratory. Percentage: proportion in each group.

^Smear negative but diagnosed as TB based on clinical symptoms and chest radiograph. TB, tuberculosis

Study 1 examined the diagnostic performance of the LAM-ELISA. First, we performed an ROC analysis using all available samples. True positives were defined as positive on MGIT, LJ, or both for MTB (*n* = 134), while true negatives were positive on neither MGIT nor LJ for MTB (*n* = 155). An ROC curve is shown in Fig B in S1 Appendix. The highest Youden’s J index was obtained at the LAM cutoff value of 6.2 pg/mL (Table C in S1 Appendix). Because this value is below the LoD and LLoQ determined using measurement precisions (see Analytic validation of the LAM-ELISA), we considered that no further modification was needed for the selected 8.5 pg/mL as the LoD and 15 pg/mL as the LLoQ. Since the LAM-ELISA is being developed as a quantitative assay, the LLoQ of 15 pg/mL was then used to analyze the data from Study 1 and Study 2. The LAM-ELISA detected all smear- and culture-positive samples (*n* = 70). In smear-negative but culture-positive samples (*n* = 58), the LAM-ELISA had a sensitivity of 50.0% (CI: 37.5%–62.5%) (Table 4) of culture (largely based on MGIT culture: 57 out of 58 culture positives were MGIT culture positive, and one was contaminated on MGIT culture but positive on LJ culture). For comparison, the Xpert MTB/RIF showed a 79.3% (CI: 67.2%–87.8%) sensitivity for MTB detection (*p* < 0.0001 versus the sensitivity from the LAM-ELISA). No false positives were detected by the LAM-ELISA in non-TB patients (Table 3) with a 100% (CI: 93.6%–100%) specificity. Among all 308 specimens, 29 were identified as NTM by culture but with only three LAM positive. This resulted in a sensitivity of the LAM-ELISA for NTM at 10.4% (3/29). Additionally, one of the three LAM-positive specimens was detected by Xpert MTB/RIF as MTB. This specimen likely contained a mixture of NTM and MTB.

**Table 4:** Sensitivity and specificity of the LAM-ELISA in Study 1.

	Category	Total	LAM-ELISA		Xpert MTB/RIF	
			Number	% (95% CI)	Number	% (95% CI)
Sensitivity	Smear positive and culture positive	70	70	100.0	NT	NT
				(94.8–100)		
	Smear negative and culture positive	58	29	50.0*	46	79.3
				(37.5–62.5)		(67.2–87.8)
Specificity	Non-TB	56	56	100.0	NT	NT
				(93.6–100)		

*p < 0.0001 versus Xpert MTB/RIF. **Abbreviations**: CI, confidence interval; ELISA, enzyme-linked immunosorbent assay; LAM, lipoarabinomannan; MTB, *M*. *tuberculosis*; NT, not tested; TB, tuberculosis.

**Fig 4:**
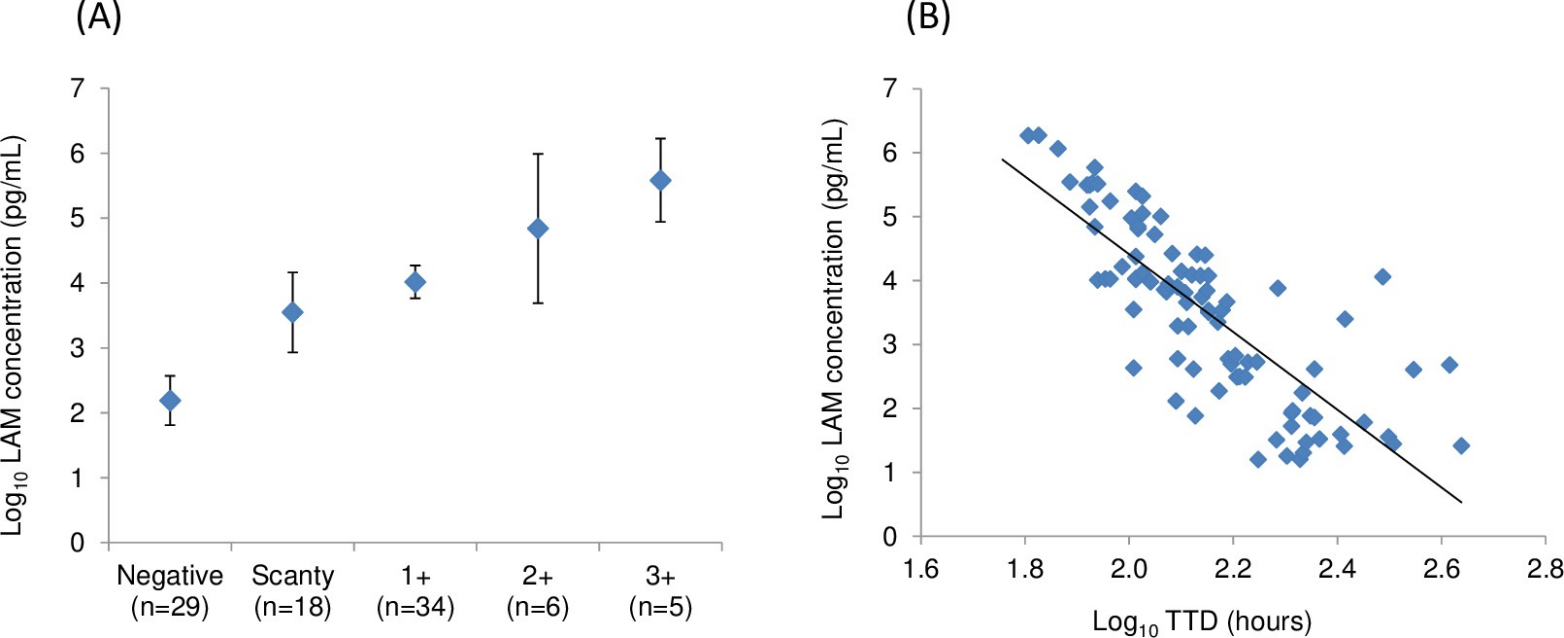
Correlation of LAM concentration with smear score or MGIT TTD in Study 1. (A) Samples were classified based on AFB smear score, and a mean value of log_10_-transformed LAM concentration of samples in each category was calculated and plotted with means ± 95% CIs. (B) Log_10_-transformed LAM concentrations and log_10_-transformed MGIT TTDs (hours) are plotted with a linear trendline. Sputum specimens with a LAM value at or above the 15 pg/mL cutoff are shown (*n* = 92). AFB, acid-fast bacillus; CI, confidence interval; LAM, lipoarabinomannan; MGIT, BACTECT MGIT 960 Mycobacterial Detection System; TTD, time to detection.

A positive linear correlation was observed between log_10_-transformed LAM concentration (pg/mL) and the AFB smear score (r = 0.721; *p* < 0.0001) in culture-positive sputum specimens using a general linear model (Fig 4A). On the other hand, the log_10_-transformed LAM concentration showed a negative linear correlation with the log_10_-transformed MGIT TTD with the following regression equation: y = −6.076x + 16.560 (*n* = 92, r = −0.803; CI: −0.714 to −0.865; *p* < 0.0001) (Fig 4B).

**Fig 5:**
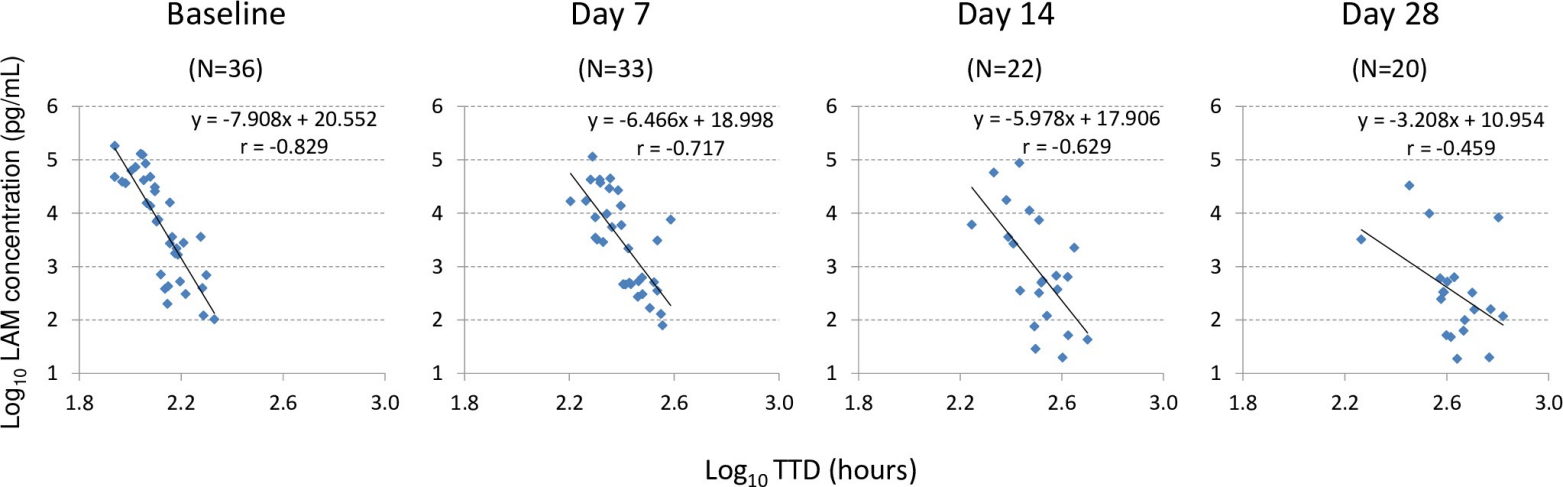
Correlation of LAM concentration and MGIT TTD at baseline, day 7, day 14, and day 28 in Study 2. Samples with valid MGIT TTDs and LAM concentrations at or above 15 pg/mL at each time point were used for the analysis. The number of samples, the regression equation, and the Pearson correlation coefficient (r) for each time point are shown in each panel. LAM, lipoarabinomannan; MGIT, BACTEC MGIT 960 Mycobacterial Detection System; TTD, time to detection.

In Study 2, we evaluated the correlation between log_10_-transformed LAM concentrations and log_10_-transformed MGIT TTDs during treatment. Only samples with a valid TTD from MGIT culture (i.e., not contaminated and confirmed as MTB complex) were included in this analysis. Log_10_-transformed LAM (pg/mL) and log_10_-transformed MGIT TTD were plotted for the baseline, day 7, day 14, and day 28 samples. Data from day 56 were not included in this analysis since most samples (19 out of 21) were negative on both the LAM-ELISA and MGIT culture at this time point. We observed a good inverse linear correlation for baseline specimens (r = −0.829; CI: −0.681 to −0.908; *p* < 0.0001), as shown in Fig 5. However, this inverse correlation relationship progressively decreased along with the increase of treatment duration with a relatively poor correlation at day 28 (r = −0.459; CI: −0.008 to −0.744; *p* = 0.041). In addition, the corresponding slopes of the linear regression lines progressively increased from −7.908 at the baseline to −3.208 at day 28.

We analyzed the number of positive cases by MGIT culture, AFB smear, the TB-LAMP, and the LAM-ELISA at each time point during treatment in Study 2. As shown in Table 5, AFB smears became negative quickly in sputum specimens from most of the patients. Note that although all patients had a positive AFB smear during screening, only 69% of patients (25 out of 36 MGIT-positive samples) were positive from the baseline in the study. In the case of MGIT culture, all samples were MTB positive (excluding contaminated samples) at the baseline and days 7 and 14. On day 28, 92% of samples were positive, but only 14% were positive on day 56. In the case of the LAM-ELISA, positive rates decreased slowly but steadily from day 7 to day 28 and were 24% at day 56.

**Table 5:** Number of positive cases by each test during treatment among sputum specimens with MGIT data in Study 2.

Test	Items	Day				
		0	7	14	28	56
MGIT culture	Positive case	36	34	26	24	3
	Positivity	100%	100%	100%	92%	14%
AFB smear	Positive case	25	6	6	2	0
	Positivity	69%	18%	23%	8%	0%
TB-LAMP	Positive case	36	34	23	23	16
	Positivity	100%	100%	88%	88%	76%
LAM-ELISA	Positive case	36	33	22	20	5
	Positivity	100%	97%	85%	77%	24%
Number of MGIT data available samples at each time point		36	34	26	26	21

Samples in Study 2 with valid MGIT data (not contaminated) at each time point were used for this analysis. **Abbreviations**: AFB, acid-fast bacillus; ELISA, enzyme-linked immunosorbent assay; LAM, lipoarabinomannan; MGIT, BACTECT MGIT 960 Mycobacterial Detection System; MTB, *M*. *tuberculosis*; TB, tuberculosis; TB-LAMP, Loopamp MTBC Detection Kit.

As shown in Table 6, the agreements between the results of the LAM-ELISA and MGIT culture at day 28 and day 56 were 85% (22/26) and 81% (17/21), respectively. At day 56, there were three specimens that were LAM positive but MGIT negative, although all had low LAM concentrations close to LLoQ (22, 23, and 78 pg/mL). On the other hand, 76% samples were positive for the TB-LAMP (16 out of 21 MGIT-data–available samples) at day 56.

**Table 6:** Number of positive cases by LAM-ELISA and MGIT culture on days 28 and 56 in Study 2.

		MGIT culture			
	Day	28		56	
	Test result	+	−	+	−
LAM-ELISA	+	20	0	2	3
	−	4	2	1	15

A 2-by-2 table provides the numbers of cases in either positive (+) or negative (−) by the LAM-ELISA or MGIT culture. **Abbreviations**: ELISA, enzyme-linked immunosorbent assay; LAM, lipoarabinomannan; MGIT, BACTEC MGIT 960 Mycobacterial Detection System.

Longitudinal changes of LAM concentration and MGIT TTD in individual patient level during the 56-day treatment were examined. We selected patients who had data from both the baseline and day 56 and at least two additional data points between for this examination. While every sputum specimen had a valid LAM concentration, only 15 patients matched this criterion for MGIT TTD. As shown in Fig C in S1 Appendix, out of the 15 patients, there is a general agreement among 14 patients that sputum LAM concentrations decreased along with MGIT TTD increases during the 56-day treatment. In fact, 11 patients became both LAM and MGIT negative after the 56-day treatment. Patient #11 appeared to be not responding as well to treatment as others: in this patient, sputum LAM decreased about one log_10_ (from 85,593 pg/mL to 8,203 pg/mL from the baseline to day 56), while MGIT TTD increased 34 hours (from 115 hours to 149 hours) (see Fig C in S1 Appendix).

When 14-day EBA was calculated, the EBA represented by the sputum LAM concentration decreased 1.29 log_10_, along with a 221 hour increase of MGIT TTD (Table 7).

**Table 7:** Changes of LAM concentration and MGIT TTD during the 14-day treatment in Study 2.

Test		Day		Difference
		0	14	
LAM-ELISA	Average ± SD	3.93 ± 0.98	2.64 ± 1.10	1.29 ± 0.97
(pg/mL, Log_10_)				(*p* < 0.0001*)
MGIT TTD	Average ± SD	131 ± 32	352 ± 90	221 ± 76
(hours)				(*p* < 0.0001*)

Twenty-three samples had MGIT TTD data at both the baseline and day 14 in Study 2 and were used for this analysis. The average of LAM concentration was calculated using the cutoff value of 15 pg/mL, i.e., if the LAM concentration was below this limit, 15 pg/mL was used to calculate the decrease. This was applied to a total of four specimens at day 14. LAM concentrations were log_10_-transformed.

*Statistical differences of the changes between day 0 and day 14 were calculated by the paired *t* test. **Abbreviations**: ELISA, enzyme-linked immunosorbent assay; LAM, lipoarabinomannan; MGIT, BACTEC MGIT 960 Mycobacterial Detection System; SD, standard deviation; TTD, time to detection.

## Discussion

We evaluated the performance of a new immunoassay, the LAM-ELISA, to quantitate LAM concentration in preclinical studies and on sputum specimens obtained from two patient cohorts in Manila, Philippines. In preclinical studies, the LAM-ELISA demonstrated an LLoQ of 15 pg/mL LAM. It detected slow-growing NTMs but without cross-reacting to common oral bacteria. In clinical studies, sputum LAM concentrations correlated with bacterial burden determined by culture prior to treatment in pulmonary TB patients. Further, changes of sputum LAM concentration during TB treatment correlated with those of bacterial burden measured by culture. These results suggest sputum LAM concentration measured by the LAM-ELISA may have the potential as a biomarker of bacterial load prior to and during treatment.

Our estimated conversion factor of 1 pg/mL of LAM to about 8.06 CFU/mL is similar to the value reported previously [11]. Therefore, the LoD and LLoQ of 8.5 pg/mL and 15 pg/mL of LAM respectively correspond to about 69 CFU/mL and 121 CFU/mL MTB bacilli. Previous studies have suggested that solid media culture has a sensitivity around 100 CFU/mL [31], indicating the LAM-ELISA may have a similar sensitivity to that of culture on solid media. However, caution should be taken in converting the sensitivity from LAM to CFU since it was only examined using one cultured MTB strain and should be further studied using spiked sputum specimens with multiple MTB strains. Tested on sputum specimens from patients prior to treatment (Study 1), this immunoassay detected all AFB-smear–positive samples and about half of AFB-smear-negative but MGIT-culture-positive samples. Unfortunately, the high contamination rate on LJ culture in Study 1 prevented a meaningful comparison of sensitivity between this assay and LJ culture.

Sputum MTB bacterial load at diagnosis (prior to treatment) is an indicator of disease severity and infectiousness and a predictor of response to treatment [31–33]. Thus, bacterial load is useful information in TB disease management. Current tests that can quantitate bacterial load include AFB smear microscopy, culture, and cycle threshold (Ct) number from quantitative polymerase chain reaction (PCR) in treatment-naive patients [4]. However, AFB smear has poor quantitative characteristics (LoD of 5,000 to 10,000 CFU/mL) and poor dynamic range (scanty to +3, with each higher scale corresponding to a 10-fold increase) [31]. The major limitation of the culture-based method is the turnaround time for results: maximally 42 days for the MGIT culture and longer (6 to 8 weeks) for the solid medium culture. CFU count on solid media is used in clinical studies during TB drug development but is rarely used in routine TB patient care because of its high demands on facility and laboratory staff. PCR methods, such as the Xpert MTB/RIF, generate a Ct that has been shown to correlate to bacterial load [34]. However, the dynamic range of Ct is still limited, as shown by van Zyl-Smit and colleagues, in which a four-log change of CFU counts (from 1.0 × 10^6^ to 1.0 × 10^2^) increased Ct from about 20 to 27.5 [35]. Our data show that sputum LAM concentration measured by the LAM-ELISA may have the potential as a bacterial load marker prior to treatment.

During treatment, available options to measure bacterial load are further reduced. AFB smear microscopy and culture have the same limitations as discussed above. PCR-based tests do not correlate with culture results because the majority of TB patients had positive results even long after sputum cultures have become negative [36]. Several reports also demonstrated the possibility of using RNA to quantitate bacterial load during treatment [37–39], but further improvement of this type of assay will be required for use in general TB laboratories because of the complexity of the measurement. For culture-based methods, MGIT TTD has been proposed as a possible surrogate marker of CFU counts on solid medium culture [27]. However, in contrast to solid medium culture, MGIT TTD is a semiquantitative measure of bacterial load reflecting not only the number of bacilli in the MGIT tube, but also the metabolic state of the bacilli, which is likely to differ prior to and during drug treatment [40,41]. Therefore, a direct translation of MGIT TTD to bacterial load may be challenging.

Our data confirm that PCR-based tests, such as TB-LAMP, are not suitable for treatment monitoring, an observation previously made for Xpert MTB/RIF [36]. But sputum LAM may be a biomarker of bacterial load during treatment based on the following observations. First, sputum LAM positivity decreased gradually along with that of MGIT culture. Second, both LAM concentration and MGIT TTD appear to show that one patient (see Fig C in S1 Appendix, patient #11) was a slow or poor responder among 40 patients. The LAM-ELISA could identify this patient in 1-2 days, while MGIT culture took more than 6 days (149 hours). The shortened time to results favoring the LAM-ELISA will be more significant for lower bacterial load samples for which MGIT TTD would be longer. Third, LAM concentration had a strong inverse correlation with MGIT TTD at the baseline and day 7 (high r-value), although r-values decreased along with the increase of treatment duration. Several reasons may account for this deterioration of correlation: reduced bacterial load at later time points under treatment or phenotypic changes of bacilli (for example, from replicating to stationary because of drug pressure) affecting MGIT TTD [40,41]. Interestingly, the slope of the correlation between sputum LAM and MGIT TTD increased in line with the increase of treatment duration. One possible explanation for this slope change is the increase of “lag time” for bacilli to grow in the MGIT tube as the treatment progresses [40,41]; therefore, the same bacterial number inoculated in the MGIT tube of a sputum specimen from a patient at an early time point during treatment would have a shorter MGIT TTD versus those obtained at later time points during treatment. This could also contribute to the changing relationships between LAM and MGIT TTD at later treatment time points and make the translation of MGIT TTD to bacterial load during treatment challenging. Lastly, sputum LAM decreased compared with an increase of MGIT TTD during the first 14 days of treatment, with similar magnitudes to log_10_-transformed CFU and MGIT TTD changes in previous EBA studies [42,43].

The antibodies used in the LAM-ELISA and the assay itself detected slow-growing NTMs. The sensitivity of the LAM-ELISA in Study 1 was around 10% for NTMs, much lower than for MTB. Further studies are needed in different clinical settings to evaluate the clinical performance of the assay for NTM detection. Because of the relatively conserved nature of the LAM structure among slow-growing mycobacteria, it is still a question as to whether a complete MTB-specific immunoassay can be developed utilizing LAM as an antigen.

In addition to using sputum, one obvious area of interest would be to evaluate the performance of the LAM-ELISA on other types of specimens such as urine. In urine specimens collected from a subset of enrolled smear-positive TB patients, we found that the LAM-ELISA had a very poor sensitivity. The three antibodies used in the LAM-ELISA were individually evaluated by Sigal and colleagues [21] against urine specimens from HIV and TB coinfected patients, and they found that the detection antibody (antibody “TB”) does not bind to LAM in urine (see Fig 1 in the Sigal paper, in which the antibody was labeled as “O-TB”). Collectively, these data suggest that while LAM is a promising target antigen for the development of TB tests, different antibody pairs will be needed to optimize detection based on specimen type. Further fundamental understanding of the structural differences of LAM in sputum and urine may help develop high performance tests.

Our studies have several limitations. The clinical studies have relatively small numbers of patients. Study 1 is a case-control cohort study, which prevented the determination of positive and negative predictive values when using LAM as a diagnostic. CFU counts on solid media, the gold standard for quantifying bacterial load, were not determined, and therefore correlations between LAM and CFU counts are not available. In addition, in Study 2, we only studied the correlations up to 56 days in patients treated with the standard 4-drug regimen. Longer treatment durations and different populations, such as multidrug-resistant and HIV-coinfected TB patients (LAM from NTM often colonized in HIV patients and other factors may negatively impact assay performance), will need to be studied to assess whether LAM can be a biomarker for treatment response on other populations and whether drug mechanisms may differentially impact LAM changes during treatment. Further, this is an initial report on a new assay that requires external validations of the findings. Finally, long-term follow-up studies are needed to determine whether the LAM biomarker can predict clinical cure.

In conclusion, we have developed antibodies binding to epitopes unique on LAM from MTB and slow-growing NTMs and shown that an ELISA constructed with these antibodies can quantitatively measure LAM in sputum with high sensitivity and specificity. The measured LAM concentration in sputum may be a biomarker of bacterial load prior to treatment and a pharmacodynamic biomarker of changes in bacterial load during TB treatment, as demonstrated in TB patients without known HIV coinfection. However, further studies are needed to examine this relationship in HIV-coinfected TB patients. Culture-based bacterial load measurements require weeks, while LAM concentration can be obtained in a matter of hours. To address the unmet need in TB clinical trials for real-time assessment of treatment response during drug development, the Critical Path to TB Drug Regimens submitted a Letter of Intent to the US Food and Drug Administration (FDA) to qualify sputum LAM as a pharmacodynamic biomarker, and the FDA has accepted sputum LAM as a biomarker into the Biomarker Qualification Program [44]. With possible improvements in the test platform to further shorten the time to results, sputum LAM could provide a real-time treatment monitoring tool for TB treatment response and support a personalized approach to TB patient treatment management.

## Supporting information

S1 ARRIVE Checklist NC3Rs ARRIVE guidelines checklist. ARRIVE, Animal Research: Reporting of In Vivo Experiments; NC3R, National Centre for the Replacement Refinement & Reduction of Animals in Research.
(PDF)

S1 STARD Checklist STARD checklist. STARD, Standards for Reporting Diagnostic Accuracy.
(DOCX)

S1 Appendix Supporting information for methods and results. (DOCX)

S2 Appendix Prespecified analysis plans for the two clinical studies. (DOCX)

S1 Data Data set for Fig 1, Fig 4, Fig B in S1 Appendix, Table 3, Table 4, and Table C in S1 Appendix. (XLSX)

S2 Data Data set for Fig 2, Fig 5, Fig C in S1 Appendix, Table 3, Table 5, Table 6, and Table 7. (XLSX)

S3 Data Data set for Fig 3. (XLSX)

S4 Data Data set for Table A in S1 Appendix. (XLSX)

S5 Data Data set for Table B in S1 Appendix. (XLSX)

## Abbreviations

AFB: acid-fast bacillus
Ara: arabinoside
Ara*f*: arabinofuranoside
ATCC: American Type Culture Collection
BCG: Bacillus Calmette–Guérin
CFU: colony-forming unit
CI: confidence interval
Ct: cycle threshold
CV: coefficient of variance
DOT: directly observed therapy
EBA: early bactericidal activity
ELISA: enzyme-linked immunosorbent assay
FDA: Food and Drug Administration
HIV: human immunodeficiency virus
ICF: informed consent form
LAM: lipoarabinomannan
LJ: Lowenstein–Jensen
LLoQ: lower limit of quantitation
LoD: limit of detection
Man: mannopyranose
MGIT: BACTEC MGIT 960 Mycobacterial Detection System
MTB: *Mycobacterium tuberculosis*
MTX: methylthio-D-xylose
MV: missed visit
NALC-NaOH: N-acetyl-L-cysteine-sodium hydroxide
NTM: nontuberculous mycobacteria
OD: optical density
PCR: polymerase chain reaction
PI-Ara4: phosphatidylinositol-capped Ara4
PILAM: inositol phosphate capped LAM
ROC: Receiver Operating Characteristic
TB: tuberculosis
TBcID: MGIT TBc Identification Test
TB-LAMP: Loopamp MTBC Detection Kit
TDF: Tropic Disease Foundation
TTD: time to detection
