# Supplementary material for: Lipoarabinomannan in sputum to detect bacterial load and treatment response in patients with pulmonary tuberculosis: Analytic validation and evaluation in two cohorts
Source: PLoS Med. 2019 Apr 12;16(4):e1002780. doi: 10.1371/journal.pmed.1002780 (PMC6461223; doi:10.1371/journal.pmed.1002780)
Supplement: S1 Appendix — (DOCX) [file pmed.1002780.s003.docx]

**S1 Appendix**

**Preparation of anti-LAM monoclonal antibody**

Monoclonal anti-LAM antibodies were generated using the phage display technology. We initially immunized animals with purified LAM but failed to isolate monoclonal antibodies. As an alternative, we used live BCG and killed *M. tuberculosis* H37Ra as immunogens. Three rabbits and two chicken were immunized subcutaneously with either 100 mg of live *M. bovis* BCG Tokyo (BCG, Japan BCG Laboratory, Japan) or killed MTB H37Ra (Beckton Dickinson, USA) with two-week interval for five times and euthanized by pentobarbital for RNA extraction from spleen cells. BCG was used because there was no evidence that BCG-LAM is significantly different from MTB-LAM, in particular to the epitope of MTX capping (personal communication; Dr. Mary Jackson; Colorado State University, US). cDNA was synthesized from total RNA and then inserted into pCANTAB5E vector (GE Healthcare, USA). The plasmid was transformed into *Escherichia coli* JM109, resulting in the generation of subtractive single-chain antibody phage library. Several phages with high affinity against purified LAM were isolated from the scFv phage library. The cDNA gene sequences of these phages were determined and inserted into pFUSEss-CHIg-rG*03 or pFUSE2ss-CLIg-rk1 vector (InvivoGen, USA). Plasmids were transfected into CHO cells to prepare for a bivalent antibody with a rabbit Fc.

**Preparation of LAM-ELISA plates**

The LAM-ELISA plates were generated by adding the two capture antibodies “S4-20” and “G3” (250 ng each per well), followed by overnight incubation at 4°C. Blocking solution (5% sorbitol, 1% bovine serum albumin in phosphate buffered saline) was then added to each well and plates were incubated for overnight at 4°C. Finally the blocking solution was discarded and the plates were dried in a desiccator for up to 24 hours. The LAM-ELISA plate was packed in a sealed silver aluminum foil storage bag and stored in a refrigerator until use.

**Fig A**. Sample processing flow. (A) For sputum specimens in Study 1. (B) For sputum specimens in Study 2.

(A)


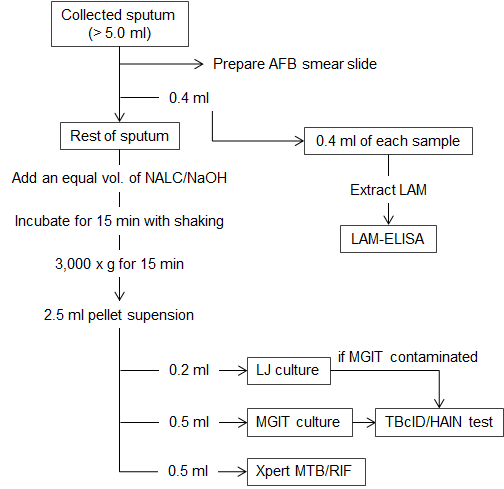


(B)


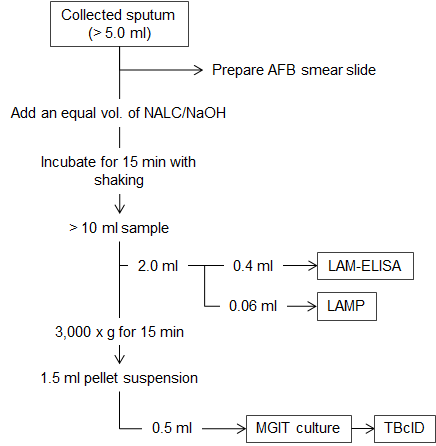


NALC/NaOH: N-acetyl-L-cysteine-sodium hydroxide solution; TBcID: MGIT™ TBc Identification Test; HAIN test: GenoType Mycobacterium CM and AS.

**Results**

**Fig B**. Receiver Operating Characteristic (ROC) analysis using Study 1 data.


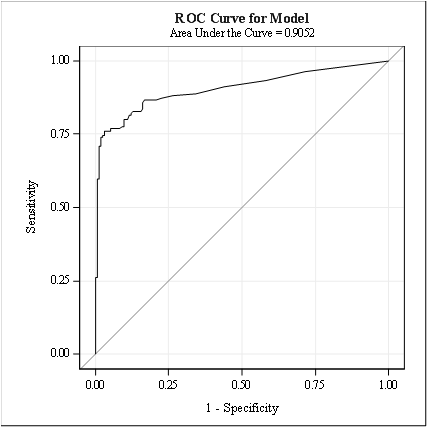


True positives were defined as those which were MGIT positive, LJ positive, or both for MTB (n=134). True negatives were defined as those which were neither positive on MGIT or LJ for MTB (n=155).

**Specificity in microbiologically negative sputum specimens from clinically-diagnosed TB patients**

The LAM-ELISA had a specificity of 97.8% (CI: 92.4-99.4%) (90 LAM negative out of 92) in microbiologically negative sputum specimens (AFB smear, MTB culture and Xpert MTB/RIF all negative) from clinically-diagnosed TB patients. There were two false positive specimens by the LAM-ELISA with relatively low LAM concentrations (15.4 pg/mL and 24.9 pg/mL); the former was determined as *M. intracellulare*.

**Fig C.** Changes of LAM concentration and MGIT TTD in fifteen patients from the Study 2.

Subjects with MGIT TTD data on both the baseline and Day 56, and with at least two additional data points between are presented. Red and blue lines show the data of LAM concentration and MGIT TTD, respectively. MGIT data below 42 days (1008 hours) and LAM concentrations below 15 pg/mL are indicated as open symbols.

To determine the effect of LAM extraction procedure on the LAM measurement, 0.4 ml of LAM solutions a at a concentration of 333 pg/ml were treated with the LAM extraction procedure (0.2 ml of 1.2 M NaOH solution and heating at 100°C for 20 min, followed by adding 0.09 ml of 5M NaH_2_PO_4_), or 0.29 ml of phosphate buffer. Three independently prepared solutions by each procedure were evaluated by the LAM-ELISA and the OD values (absorbance at 450 nm minus that at 650 nm) are shown in Table S1.

**Table A**. Effect of LAM extraction method on the readout by the LAM-ELISA.
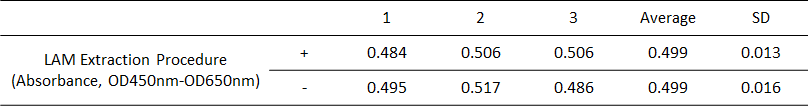


+: with the LAM extraction procedure

-: without the LAM extraction procedure

SD: Standard deviation.

**Table B.** List of tested 39 common bacteria in the oral cavity and pathogenic bacteria for pneumonia, 7 anti-TB drugs, 9 anti-pneumonia drugs and 9 anti-HIV drugs.


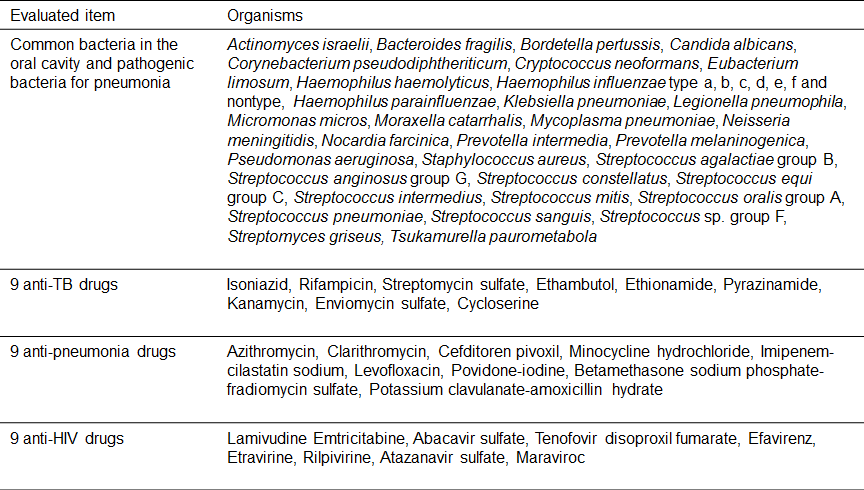


**Table C**. Youden's J Index, sensitivity, and specificity based on Study 1 data with various cutoff values.

|  | pg/mL | Highest index | Sensitivity | Specificity |
| --- | --- | --- | --- | --- |
| Optimal cutoff from Study 1 | 6.2 | 0.7289 | 0.7612 | 0.9677 |
| LoD from analytic validation | 8.5 | 0.7215 | 0.7537 | 0.9677 |
| LLoQ from analytic validation | 15.0 | 0.7130 | 0.7388 | 0.9742 |
