# Supplementary material for: Lipoarabinomannan in sputum to detect bacterial load and treatment response in patients with pulmonary tuberculosis: Analytic validation and evaluation in two cohorts
Source: PLoS Med. 2019 Apr 12;16(4):e1002780. doi: 10.1371/journal.pmed.1002780 (PMC6461223; doi:10.1371/journal.pmed.1002780)
Supplement: S2 Appendix — (DOCX) [file pmed.1002780.s004.docx]

**Prospective analysis plan**

**Study 1: (plan finalized at October 2013)**

Primary Endpoints

Performance characteristics of LAM-ELISA compare with smear and culture

1. Determine sensitivity of LAM-ELISA among smear-positive/culture positive (either LJ or MGIT) pulmonary TB patients prior to treatment
2. Determine sensitivity of LAM-ELISA in smear-negative/culture positive (either LJ or MGIT) pulmonary TB patients prior to treatment
3. Determine specificity of LAM-ELISA in smear negative, Xpert negative, and culture negative pulmonary TB suspects

Secondary Endpoints

Performance characteristics of LAM-ELISA compare with Xpert

1. Compare sensitivity of LAM-ELISA and Xpert in smear negative/culture positive (either LJ or MGIT) pulmonary TB patients prior to treatment.

Analysis of Data

Primary Endpoints

The first two endpoints estimate sensitivity of LAM-ELISA in TB patients. The sensitivity parameters will be estimated as the proportion of patients who were detected as TB positive by LAM-ELISA. For each sensitivity parameter, a 95% confidence interval will be estimated by using the normal approximation to the binomial distribution. For the third endpoint, the specificity parameter will be estimated as the proportion of TB negative patients as identified by LAM-ELISA. Again, a 95% confidence interval for the specificity parameter will be estimated by using the normal approximation to the binomial distribution.

Secondary Endpoint

The secondary endpoint compares the sensitivity of LAM-ELISA with that of Xpert. This will be performed by using the Kappa statistic as a measure of association in 2x2 tables. A 95% confidence interval will be provided for the Kappa parameter.

**Study 2: (plan finalized at January 2013)**

The LAM detection results will be compared with those from smear microscopy, LAMP, MGIT culture. Test accuracy results will be computed as sensitivity, specificity and predictive values, along with 95% confidence intervals.
